# Importance of the big-five in the future medical specialty preference

**DOI:** 10.1186/s12909-020-02151-z

**Published:** 2020-07-22

**Authors:** Jakov Milić, Ivana Škrlec, Iva Milić Vranješ, Jelena Jakab, Vera Plužarić, Marija Heffer

**Affiliations:** 1 Faculty of Medicine Osijek, Josip Juraj Strossmayer University of Osijek, Osijek, Croatia; 2 Faculty of Dental Medicine and Health Osijek, Josip Juraj Strossmayer University of Osijek, Crkvena 21, 31000 Osijek, Croatia; 3 Department of Gynecology and Obstetrics, Osijek University Hospital, Osijek, Croatia; 4 Department of Dermatology and Venerology, Osijek University Hospital, Osijek, Croatia

**Keywords:** Big-five, Medical students, Personality, Preference, Specialty

## Abstract

**Background:**

The most crucial decision in the physician’s career after graduation is undoubtedly the choice of specialization. It is conditioned by many factors such as intellectual challenges, clinical experience, economic and social influences. The aim of this study was to determine whether personality traits affect the choice of medical specialty at the University of Osijek, Croatia.

**Methods:**

This cross-sectional study included a total of 407 medical students. To assess the personality traits, the IPIP Big-Five questionnaire was used.

**Results:**

There were no differences in four of the five personality traits of the Big-Five questionnaire when comparing the groups based on their specialty preference: extroversion, agreeableness, conscientiousness, and emotional stability. A significant difference was found for openness to experience (intellect/imagination) trait, where students who preferred psychiatry specialties achieved the highest score, and those who chose public health specialties scored the lowest. We observed no significant effect between gender and specialty preference based on personality traits.

**Conclusions:**

We could not attribute the differences in personality traits to specialty preference. Medical students with higher scores on agreeableness and openness (intellect/imagination) scales were more inclined to psychiatric specialties, and more conscientiousness students preferred the anesthesiology and emergency medicine specialties. Even if variations in personality traits do not exist across different specialties, many other factors influence specialty preference.

## Background

The most crucial decision in the physician’s career after graduation is undoubtedly the choice of specialization. It is conditioned by many factors such as intellectual challenges, the possibility of professional progress, the organization of working time, salary level, but primarily the personality of a physician who makes such a decision [1–3]. Other factors which contribute to choosing a specific medical specialty are gender, economic status, clinical experience, family influence, lifestyle [4–6], and intention to work in the city [7]. Factors such as personal interests and controlled lifestyle are more important than traditional motives such as prestige and length of specialization [8]. Students are forced to decide early, usually after less than a year of short exposure to specialties during internships in a clinical setting [2]. They do not have enough experience and insights into the life and work of physicians or any specialist [2].

In European countries, including Croatia, there is a growing interest in specializations with the possibility to control one’s lifestyle. This group of specialties includes dermatology, psychiatry, radiology, ophthalmology, otorhinolaryngology, neurology, and pathology. On the other hand, on specific specializations such as anesthesiology, young doctors decide because there is too much interest in the specializations that were at the top of their priorities [1, 3].

Gender also influences the choice of medical specialty. It is well known that male students prefer specialties associated with technical and instrumental features such as surgery. While on the other hand, female students will focus on specialties with opportunities for relational aspects and more contact with patients. Gender significantly contributes to motives for specialty preferences [8, 9].

Although many factors influence a physician’s career and specialty preference, personality traits are among the most critical determinants [10]. Personality is a common intrinsic factor in determining specialty preference. Personality can be assessed in different ways and with multidimensional approaches [5]. One of the main tools to measure personality is the Big Five inventory, which consists of five personality traits: extraversion, agreeableness, conscientiousness, emotional stability/ neuroticism, and openness to experience (intellect /imagination). Extraversion includes traits such as sociability, activity, and positive emotionality. It relates to an individual’s engagement with others and the outside world. Agreeableness is related to the ability of an individual to cooperate with and to sympathize with other people. It includes traits such as altruism, tender-mindedness, trust, and modesty. Conscientiousness refers to the reliability, organization, and dutifulness of an individual. It includes being persistent, organized, and achievement oriented. Neuroticism is characterized by anxiety, anger, and depression, while emotional stability expresses ones’ capacity to remain emotionally stable and balanced under stressful circumstances. Variable ‘Openness to experience’ is often called ‘Intellect or imagination’ and it refers to the individual’s interest in the outside world and new experiences, a curiosity, sensitivity, and openness to a variety of experiences [10–12].

Different studies showed the relationship between personality traits and specialty preference. For example, psychiatrists were characterized by a lower conscientiousness [10] and high levels of openness (intellect/imagination) [5]. Psychiatrists are commonly regarded as being open to experience, especially in comparison to other medical specialties, which is consistent with previous research [5, 10, 11]. Psychiatrists are described as being imaginative, curious, intelligent, insightful, fast learning, and inventive. They thrive in their careers because of those personality traits. Psychiatrists appear to be agreeable but might vary in conscientiousness [10, 11]. In literature, agreeableness was associated with altruism, cooperation, and sympathy [4]. Psychiatrists exhibit traits associated with agreeableness such as being sympathetic, warm, trusting, helpful, cooperative, and altruistic [11]. In the literature, surgeons were characterized by extraversion [13], although different surgical specialties are characterized by different personality traits [5]. For example, students who chose gynecology and obstetrics are characterized as highly conscientious and, thus, can be described as organized, persistent, scrupulous, and achievement oriented. Also, they are less agreeable and, thus, may not be as sympathetic, friendly, or altruistic as the students that chose other specialties [11]. Anesthesiologists tend to be more extraverted and imaginative [11]. Anesthesiologists are less sociable and cooperative, but more organized, persistent, imaginative, and curious, which corresponds to extraversion and conscientiousness [11].

Although the research is consistent and showed the link between personality and academic and clinical performance, the relationship between personality and medical specialty preference is less clear [13]. It is difficult to draw a good and overall conclusion because of the variety of tools used to measure personality [4].

The present study aimed to investigate whether personality traits affect the choice of medical specialty. We hypothesized that there would be differences in personality composition concerning medical specialty preference among Osijek medical students.

## Methods

### Participants

In this cross-sectional study, we questioned a total of 407 medical students (150 males and 257 females) from the Faculty of Medicine Osijek (response rate of 81.89%). The study was conducted between January and June 2016. Students were asked to fill the questionnaire after lectures in person. The study was approved by the Ethical Committee of the Faculty of Medicine Osijek, and all participants gave written informed consent.

### Questionnaire

The students were given a self-administered questionnaire consisting of two parts. The first part included questions about the participants’ essential sociodemographic characteristics: age, grade point average (GPA) score, gender, past involvement in a scientific project, and the specialty they wish to pursue after graduation. The list of the specializations was taken from The European Union of Medical Specialists’ (UEMS) list and grouped in six fields (Table 1). In the present study, the UEMS list is used to be comparable to research in other European Union countries. The UEMS list does not offer a general practice or family medicine as a specialty. Additionaly, in Croatia it is not necessary to have a family medicine specialty to work as a family medicine physician. It is possible to work as general medicine physician right after obtaining the degree of medical doctor. Due to the similarity of family medicine and internal medicine specialty in Croatia, we could assume that most students who choose one of the internal medicine specialties might also choose family medicine. Also, in Croatia, family medicine is only a transitional period during which young physicians want to gain work experience before choosing a specialty. School success in Croatia is measured by the GPA score which applies to all levels of education. The official grade scale ranges from 1 to 5 with 1 being a failing grade, two being sufficient, three being good, four being very good, and five being excellent (Table 1).

**Table 1:** The list of medical specialties grouped into six groups

SURGERY	Surgery Thoracic Surgery Cardiothoracic Surgery Vascular Surgery Plastic, Reconstructive and Aesthetic Surgery Neurosurgery Oro-Maxillo-Facial Surgery Pediatric Surgery Orthopedics Gynecology and Obstetrics Urology Ophthalmology Otorhinolaryngology	PUBLIC HEALTH	Public Health Medicine Occupational Medicine
		PSYCHIATRY	Psychiatry Child and Adolescent Psychiatry and Psychotherapy
		ANAESTHESIOLOGY AND EMERGENCY MEDICINE	Anesthesiology Emergency Medicine
		INTERNAL MEDICINE	Internal Medicine Gastroenterology Cardiology Endocrinology Nephrology Pneumology Rheumatology Allergology Geriatrics Infectious Diseases Pediatrics Clinical Genetics Physical Medicine and Rehabilitation Radiotherapy Dermatology and Venereology Neurology
DIAGNOSTICS	Pathology Laboratory Medicine / Medical Biopathology Medical Microbiology Radiology Nuclear Medicine Clinical Neurophysiology		

In the second part, the students were asked to fill in the IPIP-50 Big Five questionnaire. Personality traits were measured with the Croatian short version of the questionnaire IPIP Big-Five with 50 items [14–16] IPIP-50 items are rated on a 5-point Likert-type scale ranging from 1 (very inaccurate) to 5 (very accurate) as in the original instrument [17].

### Statistical analysis

To test the normality of the data distribution, the Kolmogorov-Smirnov test was used. All scalar variables significantly deviated from a normal distribution. Numerical data were described with medians and interquartile ranges (IQR). To compare the means of two or more independent groups, Mann-Whitney and Kruskal Wallis tests were used, respectively. Multiple analyses of covariance (MANCOVA) were performed considering the total score of each dimension of the IPIP Big-Five as a dependent variable and taking specialty preference and gender as factors, while age was considered as a covariate to control for possible effects. The partial eta-squared (η_p_^2^) was obtained as a measure of effect size, and the observed statistical power for significant effects was > 0.90. *P* < 0.05 was considered statistically significant. The analysis was conducted using the SPSS software (ver. 16.0, SPSS Inc., Chicago, IL, USA).

## Results

We questioned a total of 407 medical students from the Faculty of Medicine Osijek. The median age of the students was 22 (IQR 20–23). Descriptive analysis is presented in Table 2.

**Table 2:** Descriptive analysis of the sample (*N* = 407)

	*n*	(%)
Gender		
Male	150	36.9
Female	257	63.1
Academic Year		
First	70	17.2
Second	84	20.6
Third	73	17.9
Fourth	5	16.0
Fifth	59	14.5
Sixth	56	13.8
Science project		
Yes	338	16.5
No	67	83.3

Of the male students, 34.7% students have selected surgical specialties, 32.7% preferred internal medicine, 12% students chose public health, 8% selected anesthesiology and emergency medicine, 7.3% students preferred diagnostics specialties, and 5.3% chose psychiatry specialties. For females, 52.9% students chose internal medicine, 21.8% preferred surgical specialties, 8.2% students selected psychiatry, 7.8% chose diagnostics specialties, 5.4% students preferred anesthesiology and emergency medicine, and 3.9% chose public health specialties.

Table 3 presents specialty preferences between groups of younger and older students.

**Table 3:** Specialty preferences between groups of younger (academic years 1–3) and older students (academic years 4–6)

	*n* (%)					
Specialty group	Years 1–3		Years 4–6		Together	
Surgery	58	(25.6)	50	(27.8)	108	(26.5)
Internal medicine	100	(44.1)	85	(47.2)	185	(45.5)
Public health	21	(9.3)	7	(3.9)	28	(6.9)
Psychiatry	16	(7.0)	13	(7.2)	29	(7.1)
Anesthesiology and emergency medicine	16	(7.0)	10	(5.6)	26	(6.4)
Diagnostics	16	(7.0)	15	(8.3)	31	(7.6)

Tables 4 and 5 present the frequencies of the students choosing a specific medical specialty, and a specialty from a specific specialty group, respectively, as well as their GPA. Since none of the students chose allergology as a preferred specialty, that specialty was excluded from Table 4.

**Table 4:** Relationship between specialty preference and gender

	*n* (%)						Median
Specialty	Male (*n* = 150)		Female (*n* = 257)		Together (*n* = 407)		GPA
Anesthesiology	11	(7.3)	14	(5.4)	25	(6.1)	4.2
Cardiology	6	(4.0)	9	(3.5)	15	(3.7)	4.0
Cardiothoracic Surgery	4	(2.7)	6	(2.3)	10	(2.5)	4.0
Child and Adolescent Psychiatry and Psychotherapy	1	(0.7)	3	(1.2)	4	(1.0)	4.0
Clinical Genetics	0	(0)	4	(1.6)	4	(1.0)	**4.5**
Clinical Neurophysiology	0	(0)	1	(0.4)	1	(0.2)	–
Dermatology and Venereology	0	(0)	15	(5.8)	15	(3.7)	4.0
Emergency Medicine	1	(0.7)	0	(0)	1	(0.2)	–
Endocrinology	4	(2.7)	14	(5.4)	18	(4.4)	4.4
Gastroenterology	1	(0.7)	2	(0.8)	3	(0.7)	4.0
Geriatrics	1	(0.7)	1	(0.4)	2	(0.5)	–
Gynecology and Obstetrics	2	(1.3)	11	(4.3)	13	(3.2)	4.0
Infectious Diseases	3	(2.0)	4	(1.6)	7	(1.7)	4.1
Internal Medicine	0	(0)	1	(0.4)	1	(0.2)	–
Laboratory Medicine/Medical Biopathology	1	(0.7)	1	(0.4)	2	(0.5)	**4.8**
Medical Microbiology	0	(0)	1	(0.4)	1	(0.2)	–
Nephrology	2	(1.3)	9	(3.5)	11	(2.7)	4.2
Neurology	**12**	(8.0)	13	(5.1)	25	(6.1)	4.0
Neurosurgery	11	(7.3)	7	(2.7)	18	(4.4)	4.4
Nuclear Medicine	0	(0)	3	(1.2)	3	(0.7)	4.0
Occupational Medicine	11	(7.3)	5	(1.9)	16	(3.9)	4.0
Ophthalmology	0	(0)	6	(2.3)	6	(1.5)	4.0
Oro-Maxillo-Facial Surgery	2	(1.3)	4	(1.6)	6	(1.5)	4.2
Orthopedics	9	(6.0)	3	(1.2)	12	(2.9)	3.9
Otorhinolaryngology	1	(0.7)	3	(1.2)	4	(1.0)	**4.5**
Pediatric Surgery	2	(1.3)	2	(0.8)	4	(1.0)	4.4
Pediatrics	**12**	(8.0)	**18**	(7.0)	30	(7.4)	4.0
Pathology	5	(3.3)	**36**	(14.0)	41	(10.1)	4.0
Physical Medicine and Rehabilitation	2	(1.3)	7	(2.7)	9	(2.2)	4.0
Plastic, Reconstructive and Aesthetic Surgery	3	(2.0)	6	(2.3)	9	(2.2)	4.2
Pneumology	0	(0)	3	(1.2)	3	(0.7)	4.4
Psychiatry	7	(4.7)	**18**	(7.0)	25	(6.1)	4.0
Public Health Medicine	7	(4.7)	5	(1.9)	12	(2.9)	3.9
Radiology	8	(5.3)	7	(2.7)	15	(3.7)	4.0
Radiotherapy	0	(0)	1	(0.4)	1	(0.2)	–
Rheumatology	0	(0)	1	(0.4)	1	(0.2)	–
Surgery	10	(6.7)	3	(1.2)	13	(3.2)	4.0
Thoracic Surgery	1	(0.7)	0	(0)	1	(0.2)	–
Urology	3	(2.0)	4	(1.6)	7	(1.7)	4.0
Vascular Surgery	7	(4.7)	6	(2.3)	13	(3.2)	4.0

**Table 5:** Relationship between specialty preference, gender and GPA

	*n* (%)						Median (IQ Range)
Specialty group	Male		Female		Together		GPA
Surgery	52	(34.7)	56	(21.8)	108	(26.5)	4.0 (4.0–4.5)
Internal medicine	49	(32.7)	136	(52.9)	185	(45.5)	4.0 (4.0–4.5)
Public health	18	(12.0)	10	(3.9)	28	(6.9)	4.0 (3.5–4.0)
Psychiatry	8	(5.3)	21	(8.2)	29	(7.1)	4.0 (3.76–4.01)
Anesthesiology and emergency medicine	12	(8.0)	14	(5.4)	26	(6.4)	4.2 (4.0–4.4)
Diagnostics	11	(7.3)	20	(7.8)	31	(7.6)	4.0 (4.0–4.5)

There was a significant difference in the preference both for a specific specialty group (Pearson Chi-Square = 25.579, df = 5, *P* < 0.001) and the individual specialties (Pearson Chi-Square = 79.965, df = 39, *P* < 0.001) between the gender.

There was a significant difference in the individual specialty preference depending on the GPA (Pearson Chi-Square = 2046.630, df = 1716, *P* < 0.001), but the difference was not observed when the specialties were merged into groups (Pearson Chi-Square = 213.525, df = 220, *P* = 0.610).

There were no significant differences in the specialty preference, depending on whether or not the students were involved with scientific research during their studies (Pearson Chi-Square = 64.45, df = 78, *P* = 0.865).

There were no differences in four of the five domains of the Big-Five questionnaire when comparing the groups based on their group specialty preference: extroversion (Kruskal-Wallis Test, *P* = 0.489), agreeableness (Kruskal-Wallis Test, *P* = 0.239), conscientiousness (Kruskal-Wallis Test, *P* = 0.367) and emotional stability (Kruskal-Wallis Test, *P* = 0.245). A significant difference was found for openness (intellect/imagination) (Kruskal-Wallis Test, *p* = 0.002). The medians and IQ-ranges of the openness (intellect/imagination) domain of the IPIP Big-Five questionnaire were as follows - surgery: 37.0 (34.0–41.0), internal medicine: 36.0 (33.0–41.0), public health: 33.5 (30.0–37.0), psychiatry: 39.0 (35–45), anesthesiology and emergency medicine: 37 (33.75–41) and diagnostics: 37 (33–40).

Females presented higher average scores in agreeableness (F_(1,394)_ = 11.17; *P* = 0.001; η_p_^2^ = 0.028), while males scored higher in emotional stability (F_(1,394)_ = 7.17; *P* = 0.008; η_p_^2^ = 0.018). Post-hoc comparisons between gender and personality traits indicated that the male students had a significantly lower agreeableness score when compared with the female students (−2.93, *P* = 0.001). Male students had a significantly higher emotional stability score when compared with female students (2.84, *P* = 0.008).

The median scores and the gender-related differences of the different domains of the IPIP Big-Five questionnaire and the students’ GPA are presented in Table 6.

**Table 6:** Relationship between personality, GPA and gender

	Male	Female	Together	
Variable	Median (IQR)			*P* value^a^
Extraversion	33 (30–39)	33 (28–37.5)	33 (29–38)	0.104
Agreeableness	36 (31–40)	39 (35–43)	38 (34–42)	**< 0.001**
Conscientiousness	35 (31–40.25)	36 (31–40)	36 (31–40)	0.832
Emotional stability	34 (28.75–37.5)	31 (27–36)	32 (27–37)	**< 0.001**
Intellect/imagination	37 (33–41)	36 (33–41)	36 (33–41)	0.458
GPA	4 (3.9–4.5)	4 (4–4.4)	4 (4–4.45)	0.753

^a^Mann-Whitney Test

Specialty preference showed significant differences in the MANCOVA for the trait openness (intellect/imagination) (F_(5,394)_ = 3.78; *P* = 0.002; η_p_^2^ = 0.046), where the psychiatry specialty achieved the highest score, and the public health specialty preference the lowest (Table 7). Post-hoc comparisons between specialty preference indicated that the students who choose public health as a specialty had a significantly lower openness (intellect/imagination) score when compared with psychiatry choice (−6.29, *P* = 0.001), surgery (−4.69, *P* = 0.003), internal medicine (−4.06, *P* = 0.011), and diagnostics (− 4.49, *P* = 0.021). We have not observed a significant effect between gender and specialty preference based on personality traits.

**Table 7:** Relationship between specialty preference and personality

	Specialty group						MANCOVA		
IPIP 50	S	IM	PH	P	AEM	D	F	η_p_^2^	*P*
Extraversion	34 (29–39)	33 (29–37)	33 (30–36)	33 (26–40)	33 (28–38)	35 (30–40)	0.752	0.009	0.585
Agreeableness	37 (33–41)	38 (34–42)	37 (33–41)	40 (37–43)	38 (34–42)	39 (36–42)	1.541	0.019	0.176
Conscientiousness	36 (21–41)	36 (32–40)	34 (29–39)	36 (32–40)	38 (32–44)	37 (32–42)	0.383	0.005	0.860
Emotional stability	33 (28–38)	31 (37–36)	32 (26–38)	32 (24–40)	32 (27–37)	33 (29–37)	0.913	0.011	0.472
Intellect/ imagination	37 (34–41)	36 (33–41)	34 (30–37)	39 (35–45)	37 (34–41)	38 (33–40)	3.779	0.046	**0.002**

Variables are defined with the median and Interquartile ranges. *S* Surgery, *IM* Internal medicine, *PH* Public health, *P* Psychiatry, *AEM* Anesthesiology and emergency medicine, *D* Diagnostics, *F* Test statistic, *η*_*p*_^*2*^ partial eta-squared, df = 5

## Discussion

The highest percentages of the students (45.5%) preferred internal medicine as a postgraduate specialty, and the lowest percentages (6.4%) preferred anesthesiology and emergency medicine. The highest level of interest was seen for internal medicine specialties. Among the subspecialties, pediatrics and neurology were the most popular, followed by endocrinology, dermatology, venereology, and cardiology. The research conducted in 2007 at the School of Medicine in Zagreb showed that the interest of students for certain specialties in Croatia had not changed much. The three most popular specialties in 2007 were internal medicine, pediatrics, and surgery [18]. Other studies conducted around the world showed that most of the students prefer internal medicine as a specialty, while the least favorite are specialties in diagnosis and psychiatry [8, 19]. Student specialty preferences may change over the course of their medical education. However, in our study there was no expected difference between students at the beginning and at the end of study regarding their specialty preferences in five groups of medical specialties (surgery, internal medicine, psychiatry, anesthesiology and emergency medicine, diagnostics). However, students at the beginning of their study showed higher interest for public health specialties, which is not in line with other studies where surgery and internal medicine are the most wanted specialties among 1st year medical students [20, 21].

New research has shown that essential factors in the specialty preference are not the same for each specialty. These differences might vary depending on the environment and even on culture. For example, in the UK, female students were less likely to choose surgical specialties [22], while in Germany, there has been a slight increase in the number of female surgeons in recent years [19]. In contrast, amongst Swedish students, there was a significant difference between females and males in the gynecology and obstetrics specialty preference [7]. In India, the male students were mostly interested in internal medicine or surgery, while females preferred gynecology and obstetrics. For females in the present study, subspecialties of internal medicine held the highest level of interest, whereas, for males, surgical subspecialties held the same place, which was expected. Females were significantly more likely than males to have an interest in pathology, endocrinology, gynecology and obstetrics, nephrology, ophthalmology, and psychiatry. Females were less likely than males to be interested in surgery, orthopedics, and occupational medicine subspecialties. There was no significant gender difference in interest in geriatrics, laboratory medicine, and radiology. Boyle and coworkers obtained similar results among medical students in New Zealand [7]. A study conducted in Switzerland showed that gender had the most significant influence on specialty preference, followed by personality traits [23].

Students who were interested in anesthesiology and emergency medicine reported the best academic performance (i.e., GPA), while those who were interested in public health reported the poorest academic performance. The crucial thing to say is that the specialty preference in Croatia is not related to income because there is no significant difference in the income across specialties, contrary to that observed in a US study [3].

There were no significant differences among specialties for extraversion, agreeableness, conscientiousness, and emotional stability traits, but there were for the openness (intellect/imagination) trait. Occupational satisfaction and personal interest are important motivating factors in specialty preferences among students who score higher at the openness (intellect/imagination) scale [4]. Openness to experience (intellect/imagination) is the most commonly reported in psychiatrists and medical students with a preference for psychiatry as a future specialty preference [24]. Openness (intellect/imagination) is associated with academic ability and divergent thinking. It is more useful in clinical education and the applied settings of medicine than in academic achievement during medical education [10]. The openness facilitates acceptance, flexibility, and adequate adaptation to situational changes [10]. Persons who score higher on openness (intellect/imagination) are more open-minded and people-oriented. They might be more intellectually curious and experience fewer obstacles to or fear of experiencing close contact with patients [24]. Of the five personality traits, openness (intellect/imagination) is the most consistent trait associated with a specialty preference, as shown in a study by Mullola et al. [10].

Females scored significantly higher than males on agreeableness but significantly lower than males on emotional stability. Agreeableness presents self-control regarding disciplined aspirations toward goals and strict adherence to personal principles [25]. In the present study, male students tend to be better at controlling their emotions, while female students tend to be more kind, helpful and sensitive toward others (Table 6). However, students pursuing diagnostics specialties scored higher on extraversion and lower on emotional stability. There is some inconsistency with other studies where females score higher on extraversion, conscientiousness, and emotional stability traits, but lower on openness (intellect/imagination) compared to males [10, 26]. The study of Mullola and coworkers found no gender differences in agreeableness [10], but Lydon and coworkers found that females scored significantly higher than males on agreeableness [13]. Our results also suggest that gender-related personality might be an essential individual-level factor in career counseling and specialty supervision during and after medical education to improve the ability of physicians to do their best.

In the present study, medical students who preferred psychiatry as a specialty showed the highest score on agreeableness and openness (intellect/imagination) traits. Although psychiatrists scored higher on extraversion in other studies [10, 11], here we found that students who choose psychiatry specialties had lower extraversion score together with students who choose internal medicine, public health, and anesthesiology and emergency medicine specialties. That trait described them as reserved, shy, silent, and retiring.

In the present study, the highest emotional stability was associated with preference in internal medicine. Many other studies found that preference in internal medicine is associated with high conscientiousness and high agreeableness [5, 10, 11]. According to our findings, we could tell that student who preferred internal medicine specialties were generally more stable, calm, and content. Previous research showed that internists are less extroverted because they are inclined to focus on the inner world of ideas rather than the community relationship.

Students who chose anesthesiology and emergency medicine showed more conscientiousness because they were highly self-reliant. Anesthesiologists had the highest conscientiousness levels, so they tend to be more organized, responsible, precise, and practical. All the traits mentioned above are essential features for anesthesiologists. Although, some studies claim that anesthesiologists are team players and open to experience [1, 13], which corresponds to high extraversion and high openness (intellect/imagination) personality traits, respectively. Being imaginative suggests that anesthesiologists could be described in the same way as surgeons concerning their imagination, curiosity, and the need for diversity.

We found that the preference for specializing in surgery is associated with high emotional stability and the lowest agreeableness. Other studies found that higher conscientiousness was associated with specializing in surgery [10] because surgeons tend to be organized, careful, and persistent. Higher emotional stability and lower agreeableness, was also associated with specializing in surgery [10]. Mainly, surgeons have been described as extraverted and outgoing [5, 10, 11]. Surgeons have a higher tendency to be demanding, dominant, and tough-minded, which refers to their lower agreeableness, and is consistent with previous research [5, 10].

Students who preferred specializations in public health scored the lowest on agreeableness, conscientiousness, and openness to experience (intellect/imagination) traits. Taken in general, these findings might suggest that public health residents tend to be more unfriendly, cruel, careless, irresponsible, shallow, and simple when compared to other specialists.

Medical students who choose diagnostic specialties scored the highest on extraversion and lowest on emotional stability. They are characterized by being more talkative, energetic, outgoing, sociable, but also anxious, fearful, and emotional [13], even though we must observe that pathologists tended to be introverted and less sociable [11]. The finding that the extroverts preferred diagnostic specialties is fascinating and requires further attention in future research.

From all of the above, we can see that the relationship between personality and medical specialty preference is not very clear and univocal.

### Limitations of the study

We questioned students from all six years of study, younger students presumably having little knowledge about the different specialties and what they entail. It is known that the specialty preferences may vary significantly from the beginning to the end of medical school. The specialties were grouped to the authors’ best ability, after consulting the literature and medical doctors from different fields. However, it was challenging to make a small number of groups without pooling in specialties that different personality groups would choose. Also, we have to take into consideration that participants will want to manage how they appear when responding to self-report questionnaires. Although the UEMS list was used so the research could be compared with studies in other European Union countries, in further studies it would be better to include family medicine as a specialty preference to gain more accurate data for Croatia.

## Conclusion

Personality traits might be helpful in medical career counseling during and after medical education to help students choose the specialty that best suits their personalities. This study showed that there were no significant differences in personality traits between medical specialty groups, except for differences in openness (intellect/imagination) among different specialty preferences. Even if variations in personality traits do not exist across different specialties, many other factors influence on specialty preference. However, we believe that our study supplements some new knowledge about the mechanism of specialty preference among medical students. Despite the limitations, the present study might be of help to medical students, professors, and medical educators in the process of selecting a specialty. Additional research with a more significant number of students will be needed to assess the more precise factors associated with the specialty preference. Our results present the influence of personality traits in Croatian settings. The present study explores the factors involved in the choice of specialty. In future studies, it would be interesting to define the most relevant personality traits necessary in each specialty.

## Data Availability

The authors have full control of all primary data and if you agree to allow the journal to review their data if requested.

## Abbreviations

GPA: Grade point average GPA
IQR: Interquartile ranges
MANCOVA: Multiple analyses of covariance
UEMS: The European Union of Medical Specialists’
